# Would You Reduce this Knee?

**DOI:** 10.5811/cpcem.2017.5.34229

**Published:** 2017-11-03

**Authors:** Mohammad R. Mohebbi, Christopher S. Sampson, Matthew T. Robinson

**Affiliations:** University of Missouri-Columbia, Department of Emergency Medicine, Columbia, Missouri

## CASE PRESENTATION

**Image 1:**
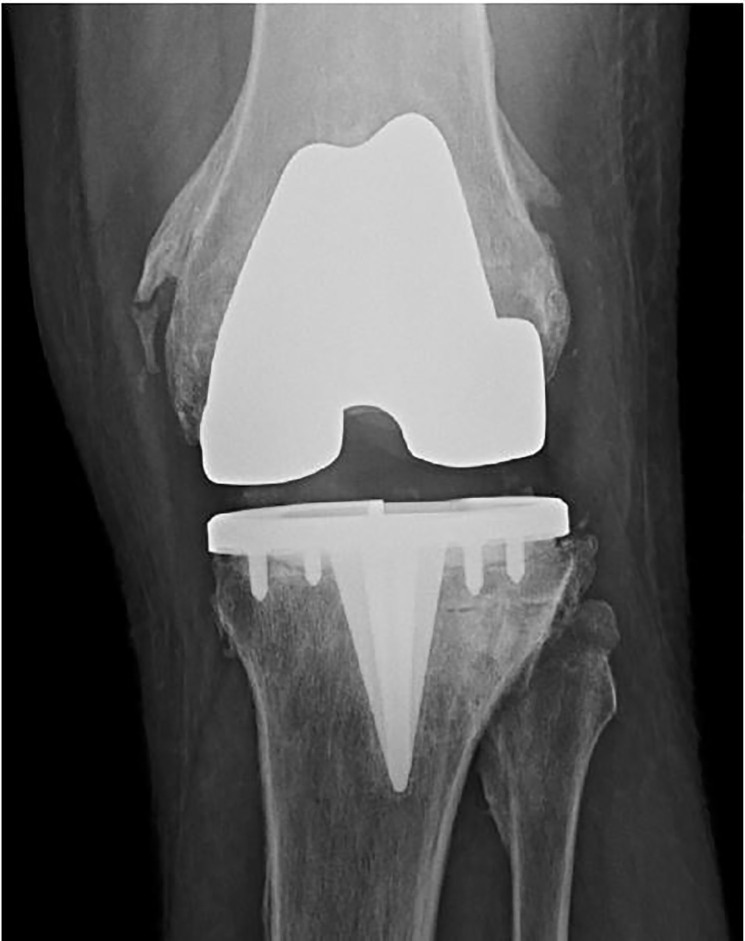
Anteroposterior radiograph of the left knee showing no evident hardware failure in a patient with history of knee replacement who presented with pain and decreased range of motion.

**Image 2:**
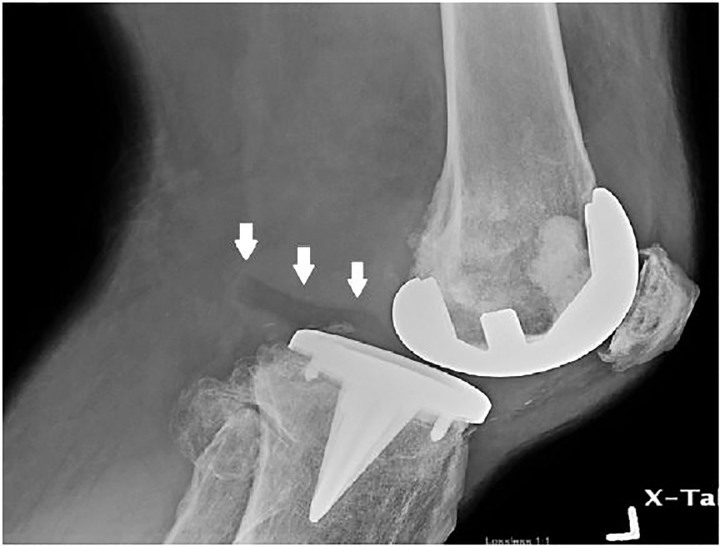
Lateral radiograph of the left knee showing posterior subluxation of the tibia with posterior displacement of the tibial polyethylene spacer (arrows).

**Image 3:**
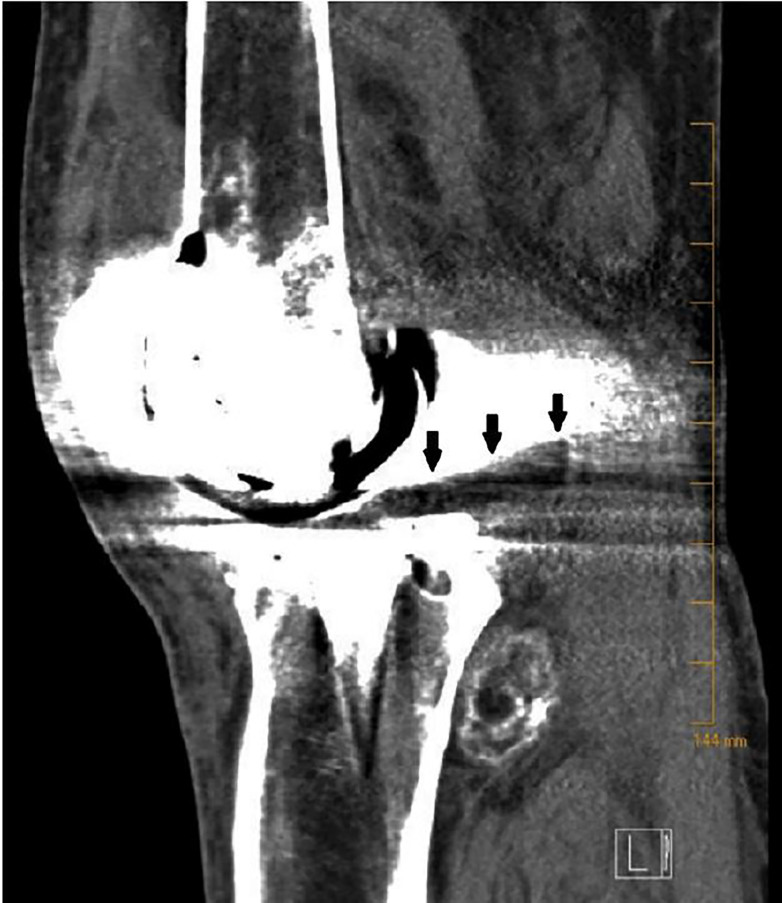
Computed tomography of left knee (sagittal view) showing posterior subluxation of the tibia with posterior displacement of the tibial polyethylene spacer (arrows).

A 46-year-old male with a history of knee replacement presented with pain and decreased range of motion of the left knee. He had felt a pop in his left knee when putting on his pants three days previously. He was standing on one leg with the weight-bearing left leg slightly flexed when the symptoms started. He had not been able to bear weight since. He was seen at a local hospital and initially diagnosed with a knee dislocation. Reduction was not carried out due to a stated allergy to ketamine, and transfer to a tertiary centre was recommended. The patient elected to go home and presented to our emergency department (ED) three days later. On physical exam, movements of the knee were severely limited. There was moderate effusion with no ecchymosis and the patient was unable to bear weight. Skin was intact. Distal pulses were palpable. Left knee radiograph showed no hardware failure in the anteroposterior view; however, the lateral view showed posterior subluxation of the tibia with polyethylene spacer unseated and displaced posteriorly (Images 1 and 2). Computed tomography provided more detail (Image 3).

## DIAGNOSIS

Dislocation of the polyethylene component of knee arthroplasty is a rare complication. The real incidence is unknown,^1^ and only a few cases have been reported.^1^ While dislocation of this component can be diagnosed on plain radiographs, it may be easily missed due to radiolucency of polyethylene. As with any knee dislocation, these injuries may be associated with injury to the popliteal vessels.^2^ Reduction attempt in the ED should be avoided due to high failure rate of a closed reduction. Our patient was admitted to orthopedics for revision of the left total knee arthroplasty.

**CPC-EM Capsule**

What do we already know about this clinical entity?

Dislocation of the polyethylene component of knee arthroplasty is a rare complication and may be associated with injury to the popliteal vessels.

What makes this presentation of disease reportable?

Only a few cases have been reported.

What is the major learning point?

Reduction attempt in the ED should be avoided due to high failure rate of a closed reduction.

How might this improve emergency medicine practice?

Orthopedic consult should be done as reduction attempt is often unsuccessful and may cause complications. Revision of the arthroplasty is the treatment of choice.
